# *miR‐22* **enhances the radiosensitivity of small‐cell lung cancer by targeting the** *WRNIP1*

**DOI:** 10.1002/jcb.29032

**Published:** 2019-06-12

**Authors:** Wenhua Jiang, Xuemei Han, Jingrui Wang, Lin Wang, Zanmei Xu, Qiao Wei, Wenpan Zhang, Haitao Wang

**Affiliations:** ^1^ Department of Radiotherapy Tianjin Medical University Second Hospital Tianjin China; ^2^ Department of Respiration Tianjin Medical University Second Hospital Tianjin China; ^3^ Tianjin Marvelbio Technology Co, Ltd Tianjin China

**Keywords:** DEGs, *miR‐22*, radiosensitivity, SCLC, *WRNIP1*

## Abstract

Small‐cell lung cancer (SCLC) is an aggressive malignancy characterized by high cellular proliferation and early distant metastasis. Our study aimed to explore the effect of *miR‐22‐3p* (*miR‐22,* for short) on SCLC radiosensitivity and its molecular mechanisms. The expression level of *miR‐22* was evaluated in a human normal lung epithelial cell line and a human SCLC cell line, and cell apoptosis and migration were detected. The expression of the *miR‐22* direct target *WRNIP1* mRNA and protein were explored. Five differentially expressed genes were detected. The *miR‐22* expression in NCI‐H446 was significantly decreased, and *miR‐22* overexpression significantly promoted cell apoptosis. *miR‐22* overexpression could significantly inhibit the cell migration of SCLC cells, and *miR‐22* had a negative regulatory effect on *WRNIP1* mRNA and protein levels. *KLK8* was downregulated, and the messenger RNA (mRNA) of four other genes (*PC, SCUBE1, STC1*, and *GPM6A*) was upregulated mRNA in cells overexpressing *miR‐22*, which was in accordance with the bioinformatics analysis. *miR‐22* could enhance the radiosensitivity of SCLC by targeting *WRNIP1*.

## Graphical abstract

1. *miR‐22* overexpression strongly promoted small‐cell lung cancer (SCLC) cell apoptosis upon γ‐ray treatment. 2. *miR‐22* overexpression can significantly inhibit the cell migration of SCLC cells under γ‐ray treatment. 3. *miR‐22* is a direct negative regulatory of WRNIP1. 4. The differentially expressed gene (DEG) *KLK8* downregulated messenger RNA (mRNA) and other four DEGs upregulated mRNAs in *miR‐22* overexpression.

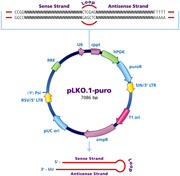

## 1 INTRODUCTION

Lung cancer (LC) has the highest morbidity and mortality in the world, which seriously threatens human life and health.^1^ Small‐cell lung cancer (SCLC) is the main type of LC, which has the characteristics of short multiplication, rapid growth, and early‐onset metastasis.^2^ With the rapid development of stereotactic radiosurgery and radiotherapy technology, radiotherapy provides an effective strategy for SCLC treatment. A growing number of studies have revealed that SCLC is sensitive to radiotherapy. SCLC is prone to relapse, resulting in radioresistance in the late stage of radiotherapy; thus the effect of radiotherapy is regrettably reduced.^3^ Therefore, there is an urgent need to fully elucidate the therapy‐induced radioresistance mechanisms to improve the radiotherapy effect and prolong the survival of patients with SCLC.^4^

Ionizing radiation (IR) is one of the major modalities of SCLC treatment.^5^ It causes DNA damage by producing intermediate ions and oxygen free radicals, leading to tumor cells apoptosis.^6^ Although the DNA damage pathway plays an important role in radiation sensitivity, cell cycle checkpoint, and apoptosis pathways also play important roles in the susceptibility of SCLC to radiotherapy. Recently, scientists have devoted themselves to discover how IR attacks SCLC cells.^7^ Disappointingly, the relevant mechanisms remain obscure. SCLC radiotherapy is limited and ineffective due to SCLC radioresistance.^8^ Therefore, to explore effective and specific methods to enhance the radiosensitivity of SCLC is helpful to reduce radioresistance and side effects.^9^, ^10^

An increasing number of studies show that microRNAs (miRNAs) act as gene expression regulators in tumor initiation and procession, which cause translation inhibition by messenger RNA (mRNA) inactivation and degradation.^11^, ^12^ As negative regulators, miRNAs can act on essential signaling pathways, including cell response to IR.^13^ Therefore, regulating the expression of miRNAs has a significant impact on the clinical radiation response, as it enhances cell susceptibility.^14^ Although no miRNAs have been approved by the Food and Drug Administration as drugs, greater progress is being made in registering them as therapeutic agents.^6^ Moreover, the latest research has demonstrated that the efficacy of miRNA‐based therapeutic agents in preclinical models.^15^

Recently, *miR‐22‐3p* (*miR‐22*, for short), which is a 22 nucleotide noncoding RNA located on chromosome 17, has been found to regulate tumor‐related gene expression in different cancer models.^16^ However, whether *miR‐22* controls tumor radioresistance in vivo and can be used as a tumor radiosensitizer remains unclear. In this study, we find the *miR‐22* has significant effects on SCLC cell proliferation, migration, and apoptosis in an IR dose‐dependent manner. We further show that *WRNIP1* is a direct target of *miR‐22*, and the related molecular mechanism of *miR‐22* regulation of SCLC radiosensitivity is preliminarily explained. More importantly, our findings demonstrate the therapeutic utility of *miR‐22* as a potential tumor radiosensitizer in a SCLC model. These results suggest that the *miR‐22* cargo combined with radiotherapy may represent a new strategy for SCLC treatment.

## 2 MATERIALS AND METHODS

### 2.1 Cell culture

Cell lines were used in this paper. Human normal lung epithelial cell line BEAS‐2B, human SCLC cell line NCI‐H446 and human embryonic kidney cell line HEK293T were purchased from the Cell Bank of Shanghai Institute of Cell Biology, CAS. The cells were cultured in high glucose Dulbecco's modified Eagle's medium and Roswell Park Memorial Institute 1640 (Gibco, UK) supplemented with 10% fetal bovine serum (FBS; Gibco) and 1% penicillin (100   U/mL)‐streptomycin (100   μg/mL; Gibco) and were maintained in a 37°C incubator with a humidified, 5% CO_2_ atmosphere.

### 2.2 Cell transfection

Cells were transfected with vector controls and miRNA compounds by Lipofectamine 2000 (Invitrogen), according to the manufacturer's protocol. After 24 to 48 hours of transfection, cell samples were collected and subjected to transfection‐efficiency testing.

### 2.3 *miR‐22* mimics/nc and inhibitors/nc oligonucleotides

Four individual products (GenePharma, China) were synthesized (Table 1). Cells were transfected with 100 nM of the indicated oligonucleotide separately using Lipofectamine 2000. Then, 24 to 48 hours after transfection, the resultant cells were used for functional assays, and the remaining cells were harvested for quantitative polymerase chain reaction (qPCR) analysis**.**

**Table 1:** The oligo sequences of *miR‐22* mimics and inhibitors

Name	Sequence (5′‐3′)
*miR‐22*‐mimics	AAGCUGCCAGUUGAAGAACUGU
	AGUUCUUCAACUGGCAGCUUUU
*miR‐22*‐mimics nc	UUCUCCGAACGUGUCACGUTT
	ACGUGACACGUUCGGAGAATT
*miR‐22*‐inhibitors	ACAGUUCUUCAACUGGCAGCUU
*miR‐22*‐inhibitors nc	CAGUACUUUUGUGUAGUACAA

### 2.4 Overexpression cell construct

**Figure 1:**
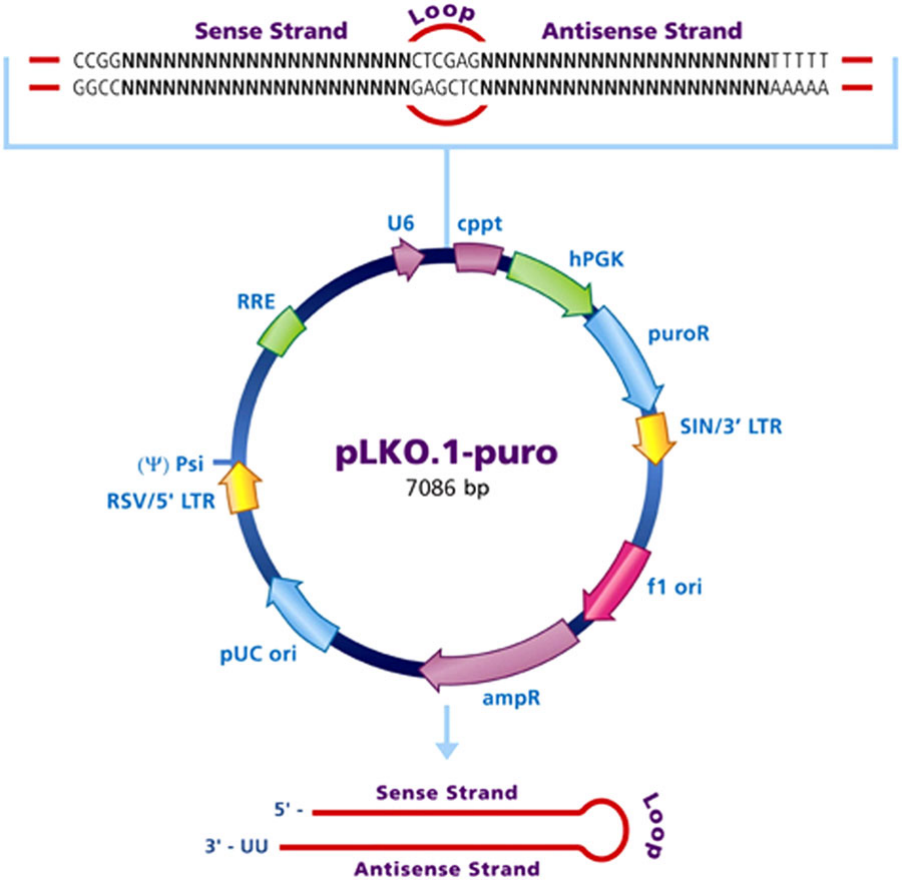
The map of pLKO.1 vector

The *miR‐22* oligos were cloned into the pLKO.1 vector according to the manufacturer's instructions (Figure 1). The cells were transfected with the pLKO.1 control and *miR‐22*‐pLKO.1 plasmids, followed by drug screening and qPCR analysis. The primer sequences are shown in Table 2.

**Table 2:** miRNAs stem‐loop primer sequences

Name	Sequence (5′‐3′)
*miR‐22* stem‐loop primer	GTCGTATCCAGTGCAGGGTCCGAGGTATTCGCACTGGATACGACACAGTT
*U6* stem‐loop primer	GTCGTATCCAGTGCAGGGTCCGAGGTATTCGCACTGGATACGACAAAATA

Abbreviation: miRNAs, microRNAs

### 2.5 RNA extraction and real‐time PCR analysis

Total RNA was extracted using the RNAiso Plus Kit (TaKaRa, Japan). For complementary DNA (cDNA) synthesis, total RNA was involved in the cDNA amplification by the HiScriptII Reverse Kit (Vazyme, China), and for qPCR analysis, the AceQ real‐time (RT)‐qPCR Kit (Vazyme) was used, all according to the manufacturer's instructions. The mRNA levels were normalized by *U6* and *β‐actin*. The primer sequences are shown in Table 3.

**Table 3:** qPCR primer sequences

Name	Sequence (5′‐3′)
*miR‐22*‐F	AGGGTCCGAGGTATTCGCA
*miR‐22*‐R	AGCGAAGCTGCCAGTTGAAG
*U6*‐F	CTCGCTTCGGCAGCACA
*U6*‐R	AACGCTTCACGAATTTGCGT
*WRNIP1*‐F	ATTGATGAGATTCATCGGTTCAA
*WRNIP1*‐R	GGCTAGAGTCTAGGACGTGGATTC
*β‐Actin*‐F	CTGGACTTCGAGCAAGAGAT
*β‐Actin*‐R	GATGTCCACGTCACACTTCA
*GPM6A*‐F	GTTTATTGTGGCACTTGCTGGA
*GPM6A*‐R	TGGCAGACAGAACCATAAGGTAGTG
*STC1*‐F	AAATGCATCGCCAACGGG
*STC1*‐R	TTCATCACATTCCAGCAGGCTT
*KLK8*‐F	GAAGTGTGAGGATGCTTACCCG
*KLK8*‐R	ATGTGATGCCCTGGAGTGC
*PC*‐F	CTGCGGTCCATCTTGGTCAA
*PC*‐R	CCATGGGTGAGGTCACCAC
*SCUBE1*‐F	AACTCATAGAGGACATCGTGCG
*SCUBE1*‐R	CGCTCCCCCCGGTTATTT

Abbreviations: F, forward; qPCR, quantitative polymerase chain reaction; R, reverse.

### 2.6 Lentivirus preparation

The cells were inoculated into six‐well plates at a density of 2 × 10^6^ cells per well and were cultured overnight in an incubator at 37°C. The cells were infected with pLKO.1 vector control or *miR‐22*, carrying lentiviral particles for 48 hours. After selection with 10 μg/mL puromycin, overexpression efficiency was verified by qPCR analysis.

### 2.7 Western blotting

Cells were collected and resuspended in 0.5 mL ice‐cold radioimmunoprecipitation assay lysis buffer (Solarbio, China). Cell lysates were lysed by vortexing with acid‐washed glass beads for 2 minutes, then placed on ice for 2 minutes; this cycle was repeated 10 times. After centrifugation at 12 000 rpm for 30 minutes, the sample protein concentration was determined using Nanotrop (Thermo Fisher Scientific). Lysate samples (50 μg) were electrophoresed on 8% or 10% polyacrylamide gels and transferred onto polyvinylidene difluoride membranes. The samples were blocked with 5% nonfat milk in Tris buffered saline solution containing 0.1% to 0.2% Tween‐20 at room temperature for 2 hours and then probed with primary antibodies (anti‐WRNIP1, 1:1000 and anti‐β‐actin, 1:1000; Santa Cruz), as indicated, overnight at 4°C; the samples were then incubated with horseradish peroxidase‐conjugated secondary antibodies (anti‐mouse, 1:2000; Santa Cruz). The bands were visualized using an ECL chemiluminescence detection kit (Thermo Fisher Scientific). Multiplication of the intensity and area of protein bands indicated the relative levels of protein expression.

### 2.8 γ‐Ray treatment

The prepared cells were divided into three parts and sent to the Institute of Radiological Medicine of the Chinese Academy of Medical Sciences for γ‐irradiation with doses of 0, 2, and 4 Gy, respectively.

### 2.9 *miR‐22* expression levels

qRT‐PCR was performed to detect the *miR‐22* expression levels in cells after γ‐irradiation. The experimental method was the same as in section 2.5.

### 2.10 3‐(4,5‐dimethylthiazol‐2‐yl)‐ 5‐(3‐carboxymethoxyphenyl)‐ 2‐(4‐sulfophenyl)‐2H‐tetrazolium assay

The transfected cells were detected by using the CellTiter 96 AQ MTS Reagent Powder kit (Promega), according to the manufacturer's protocol. The 3‐(4,5‐dimethylthiazol‐2‐yl)‐5‐(3‐carboxymethoxyphenyl)‐ 2‐(4‐sulfophenyl)‐2H‐tetrazolium, inner salt (MTS) activity was determined by measuring absorbance at 490 nm.

### 2.11 Colony formation assay

In general, the concentration of 1 × 10^3^ cells was inoculated into six‐well plates and gently shaken in the dish in a cross direction to disperse the cells evenly. The cells were cultured in a 37°C incubator with a humidified, 5% CO_2_ condition for 7 to 10 days. The cells were stained with Giemsa for 10 to 15 minutes and images were obtained. Colonies consisting of 100 or more cells were counted. The survival fraction was calculated as the mean number of colonies/(cells seeded × plating efficiency).

### 2.12 Flow cytometry analysis

Cell apoptosis was detected using an Annexin V/propidium iodide (PI) staining kit (Biolegend), according to the manufacturer's protocol. Cell proliferation was detected using a PE Mouse Anti‐Human Ki‐67 Set kit (BD Biosciences), according to the manufacturer's protocol. The cell cycle was detected using PI (50 μg/mL; Sangon Biotech, China) staining. The cells were predisposed to ethanol fixation and RNase A treatment.

### 2.13 Site‐directed mutation

**Figure 2:**
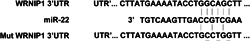
Site‐directed mutation of WRNIP1‐3′‐UTR‐psiCHECK2 mutant recombinant plasmid. 3′‐UTR, 3′‐untranslated region

The cDNA templates were changed to the locus of *miR‐22* and *WRINP1‐* 3′‐untranslated region (3′‐UTR), according to TargetScan (http://www.targetscan.org) and PICTAR (http://www.pictar.mdc‐berlin.de) predictions (Figure 2). The *WRINP1*‐ 3′UTR sequence is CTGGCAGCT, which binds to *miR‐22*. The mutation sequence is CTGCCTGGT, according to the mutation sites; primers were designed, as shown in Table 4, followed by *WRNIP1*‐3′UTR‐Mut‐psiCHECK2 construction and DNA sequencing.

**Table 4:** *WRNIP1*‐3′‐UTR‐Mut primer sequences

Name	Sequence (5′‐3′)
*WRNIP1*‐3′‐UTR‐mut‐F	AAAATACCTGCCTGGTTTGTGCAATGAATTAATGT
*WRNIP1‐*3′‐UTR‐mut‐R	CAAACCAGGCAGGTATTTTCATAAGCATAACCG

Abbreviation: 3′‐UTR, 3′‐untranslated region.

### 2.14 Transcriptome sequencing

The *miR‐22* overexpressing SCLC cell line NCI‐H449 stably transfected *miR‐22*‐NCI‐H449 and the empty control pLKO.1‐NCI‐H446 were sequenced. The cells were cultured to a concentration of 1 × 10^7^, which met the sequencing concentration requirement. The confirmatory samples were sent to Novegene Biotechnology Co, Ltd for transcriptome sequencing. The sequencing data generated was 5 Gb per sample. Bioinformatics analysis was carried out according to the raw reads and clean reads.

### 2.15 Luciferase assay

The cells were seeded in 96‐well plates at a density of 5 × 10^4^ per well. Luciferase activity was detected using a Dual‐Luciferase Reporter Assay System kit (Promega), according to the manufacturer's protocol. A microplate reader was used to determine relative luminescence.

### 2.16 Bioinformatics analysis

The human genome and vector control were considered as the reference genome and control sequence, respectively. The expression abundance of the corresponding clean reads gene in the samples was obtained, and the differentially expressed genes (DEGs) were found. Then, the obtained DEGs were analyzed by Gene Ontology and Kyoto Encyclopedia of Genes and Genomes.

### 2.17 Statistical analysis

All results of this study were presented as the mean ± SEM, and statistical significance was examined by unpaired two‐tailed Student *t* test. The *P* < .05 was considered as statistically significant and indicated with *, *P* < .01 = **and *P* < .001 = ***.

### 2.18 Data availability

The authors declare that all the data supporting the findings of this study are available within the article and from the corresponding author on reasonable request.

## 3 RESULTS

### 3.1 The establishment of *miR‐22* overexpression and knock down models

**Figure 3:**
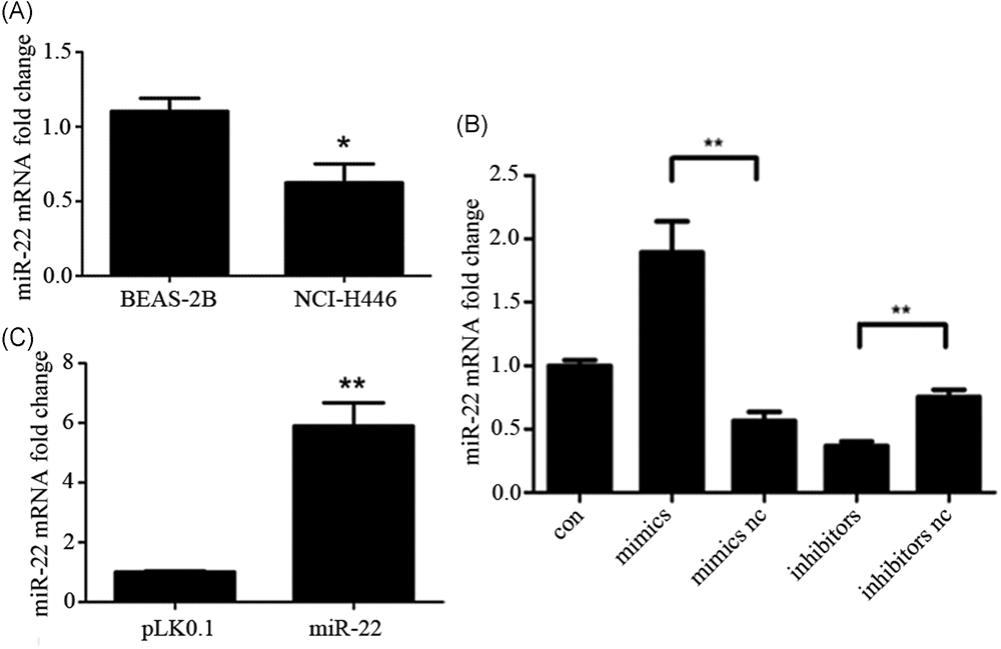
The establishment of *miR‐22* overexpression and knockdown models. A‐C, Cells were cultured in the corresponding medium for the indicated times. Efficiency was determined by quantitative PCR and measured as the ratio of *miR‐22* relative to the internal reference target *U6* gene. *,**,***Significant differences between the tested and the control strains, *P* < .05, *P* < .01, and *P*  <  .001, respectively. PCR, polymerase chain reaction

To investigate the possible function of *miR‐22* in the regulation of SCLC, we first examined the mRNA expression level of *miR‐22* in cell lines BEAS‐2B and NCI‐H446 by qPCR. Interestingly, the *miR‐22* expression was significantly decreased in the SCLC cell line (Figure 3A). Presumably, it is possible to associate *miR‐22* expression with SCLC treatment.

To confirm our hypothesis, we established the *miR‐22* overexpression and knockdown models. As a result, the transfected cells carrying *miR‐22* mimics/nc and inhibitors/nc are considered ideal tools for *miR‐22* research. As shown in Figure 3B, the *miR‐22* expression is apparently higher than the vector control upon the transfection of mimics. Otherwise, once we transfected the *miR‐22* inhibitors, the *miR‐22* expression was expectedly lower than that in the other group, indicating that *miR‐22* mimics and inhibitors have a remarkable effect on *miR‐22* overexpression and knockdown. Meanwhile, the stable *miR‐22* overexpressed cell line was successfully created by lentivirus infection and drug screening (Figure 3C).

### 3.2 *miR‐22* enhances radiosensitivity by targeting tumor development

**Figure 4:**
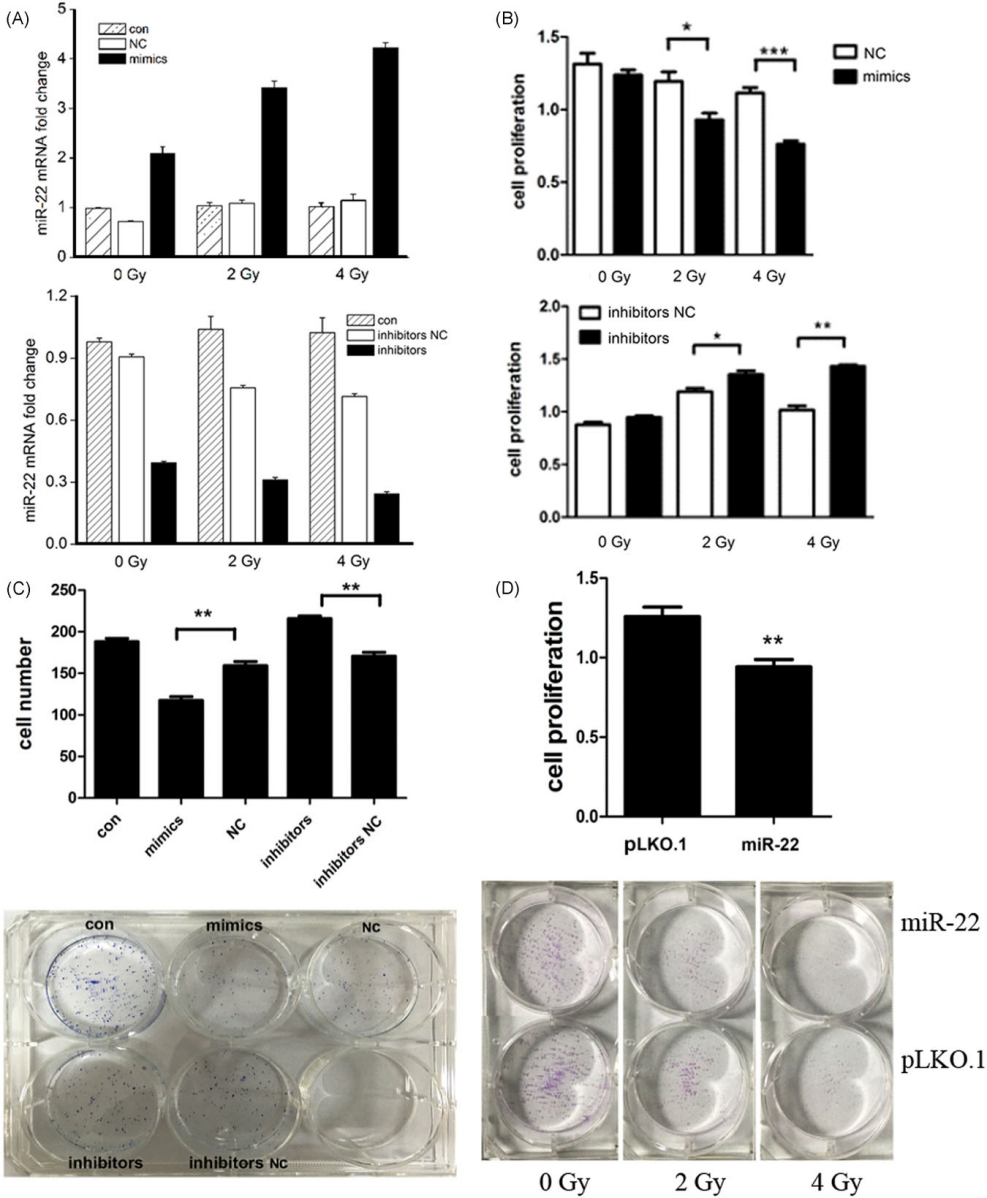
*miR‐22* inhibits the SCLC cells proliferation under different doses of γ‐irradiation. A, *miR‐22* expression in cells were detected by qPCR. B, Cells were stimulated with the indicated concentrations of MTS for the indicated conditions, cell viability was determined by microplate reader detection. CB‐DC, Cells were cultured in 96‐well plates for the indicated conditions, cell number was determined by Giemsa staining. *,**,***Significant differences between the tested and the control strains, *P* < .05, *P * < . 01, and *P*  < . 001, respectively. MTS, 3‐(4,5‐dimethylthiazol‐2‐yl)‐ 5‐(3‐carboxymethoxyphenyl)‐ 2‐(4‐sulfophenyl)‐2H‐tetrazolium; NC, negative contriol; qPCR, quantitative polymerase chain reaction; SCLC, small‐cell lung cancer

Recently, cancer cells, which are defined operationally as tumor‐initiating and tumor‐inducing cells, have been found to enhance radioresistance during DNA damage response activation.^17^ Therefore, we suspected that this *miR‐22* may be negatively associated with radioresistance in SCLC. Then, we detected the effect of *miR‐22* expression levels and *miR‐22* on the cell proliferation of NCI‐H446 under different doses of γ‐irradiation. Indeed, with the increasing dose of γ‐irradiation, the *miR‐22* expression was significantly increased in *miR‐22* mimic‐transfected cells (Figure 4A), accompanied by the inhibition of cell proliferation in the *miR‐22* overexpression group compared to the control. The proliferation level of *miR‐22* mimic‐transfected cells was lower than in the NC group during 4 Gy irradiation. Conversely, cell proliferation in the *miR‐22*‐knockdown was notably promoted and the proliferation of *miR‐22* inhibitors was higher than that under the same condition. It was concluded that *miR‐22* expression may affect the sensitivity of SCLC cells to γ‐rays and play an important role in the radiosensitivity of SCLC (Figure 4B).

In previous results, we found that *miR‐22* caused significant inhibition of SCLC cell proliferation. Afterward, we studied the effects of *miR‐22* on cell growth and colony formation by colony‐formation assay. The results revealed that the number of *miR‐22* mimic‐transfected cells was significantly lower than in the NC group. In addition, after transfecting *miR‐22* inhibitors, the number of cells was significantly higher than in the inhibitors NC group, and this result was basically consistent with the MTS results. In addition, we found the cell colony in mimics transfection was significantly less than in the NC group; transfected inhibitors were the opposite (Figure 4C). Thus, *miR‐22* inhibited colony formation in NCI‐H446.

We further investigated the cell colony‐forming ability under γ‐irradiation conditions using the *miR‐22* overexpressed cell line. Consistent with the previous analysis, the number of *miR‐22* cells was lower than control cells in the condition of γ‐ray irradiation, and the trend was enhanced with the increasing γ‐ray irradiation dose (Figure 4D). These results showed that *miR‐22* inhibited SCLC cell growth and increased the irradiation sensitivity to γ‐rays.

**Figure 5:**
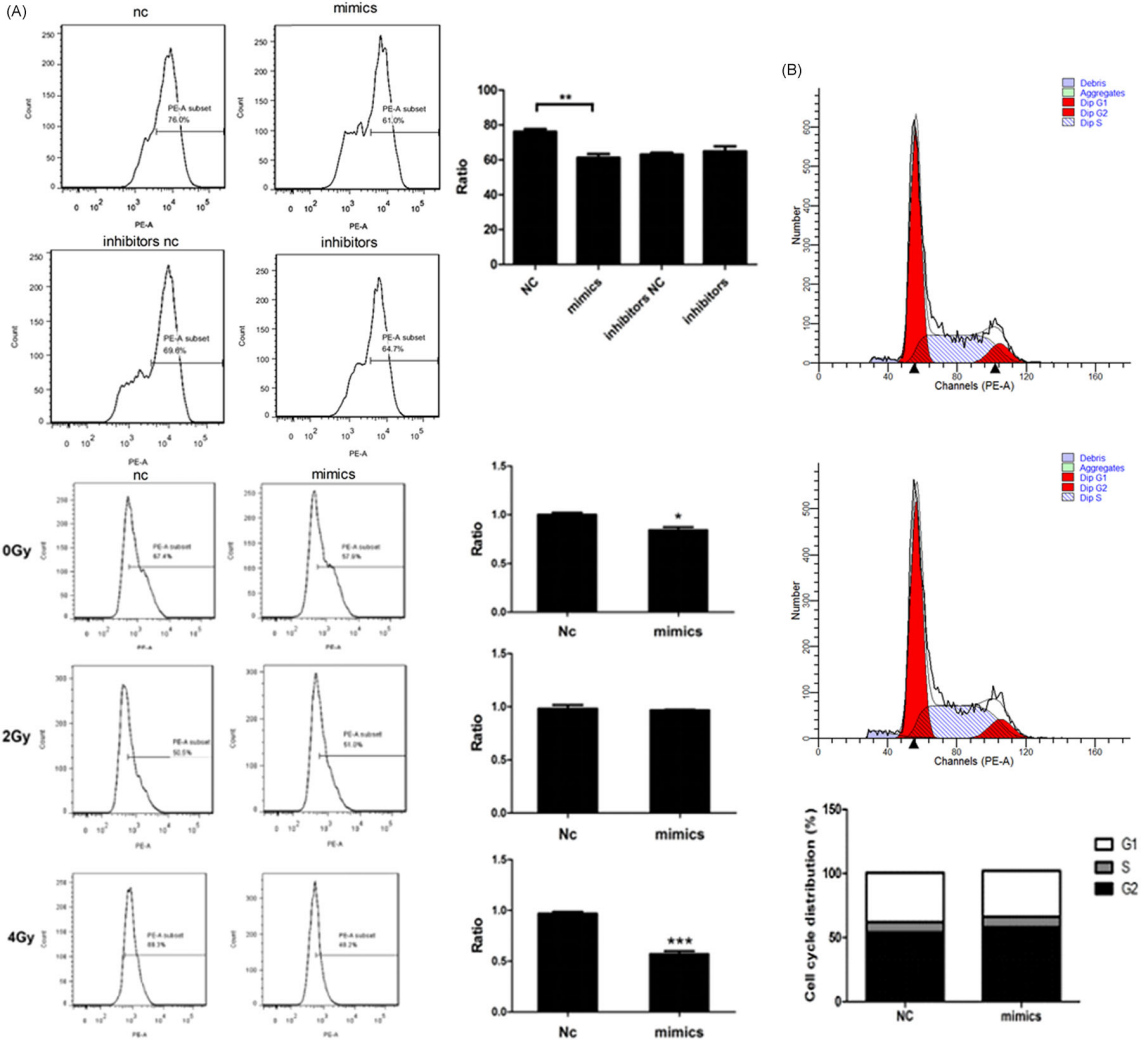
*miR‐22* affects the *Ki‐67* expression upon γ‐irradiation (A, B) Cells were cultured in 96‐well plates for the indicated conditions, Ki‐67 expression radio, and cell cycle was determined by flow cytometry analysis. *,**,***Significant differences between the tested and the control strains, *P* < .05, *P*  <  .01, and *P*  <  .001, respectively. NC, negative control

Since *miR‐22* expression may affect the mitosis‐related *Ki‐67* expression in SCLC cells,^18^, ^19^ we further investigated *Ki‐67* expression after transfection with the *miR‐22* mimics. At first, we detected *Ki‐67* expression in transfected cells under nonirradiation conditions. The results are shown in Figure 5A. After mimics transfection, the positive rate of *Ki‐67* was 61.0%. The NC transfection group showed a significant decrease. However, there was no significant difference compared to the inhibitors NC group. It was primarily shown that *miR‐22* overexpression could inhibit *Ki‐67* expression in SCLC samples.

We explored *Ki‐67* expression in *miR‐22* overexpressed cells under different doses of γ‐irradiation. The data showed that, with an increasing dose of γ‐ray irradiation, the expected decreasing *Ki‐67* expression gradually increased in the *miR‐22* overexpression group. During 4 Gy irradiation, the positive rate of *Ki‐67* in *miR‐22* overexpressed cells was 48.2%, which was lower than that in control. We inferred that *miR‐22* could inhibit *Ki‐67* expression in NCI‐H446, leading to a boycott of cell proliferation, and this inhibition was more pronounced under high dose radiation conditions. Compared with the NC control, there was no significant difference in the proportion of the cell cycle after transfection with the *miR‐22* mimics, indicating that the *miR‐22* expression level may not affect the cell cycle of SCLC (Figure 5B).

**Figure 6:**
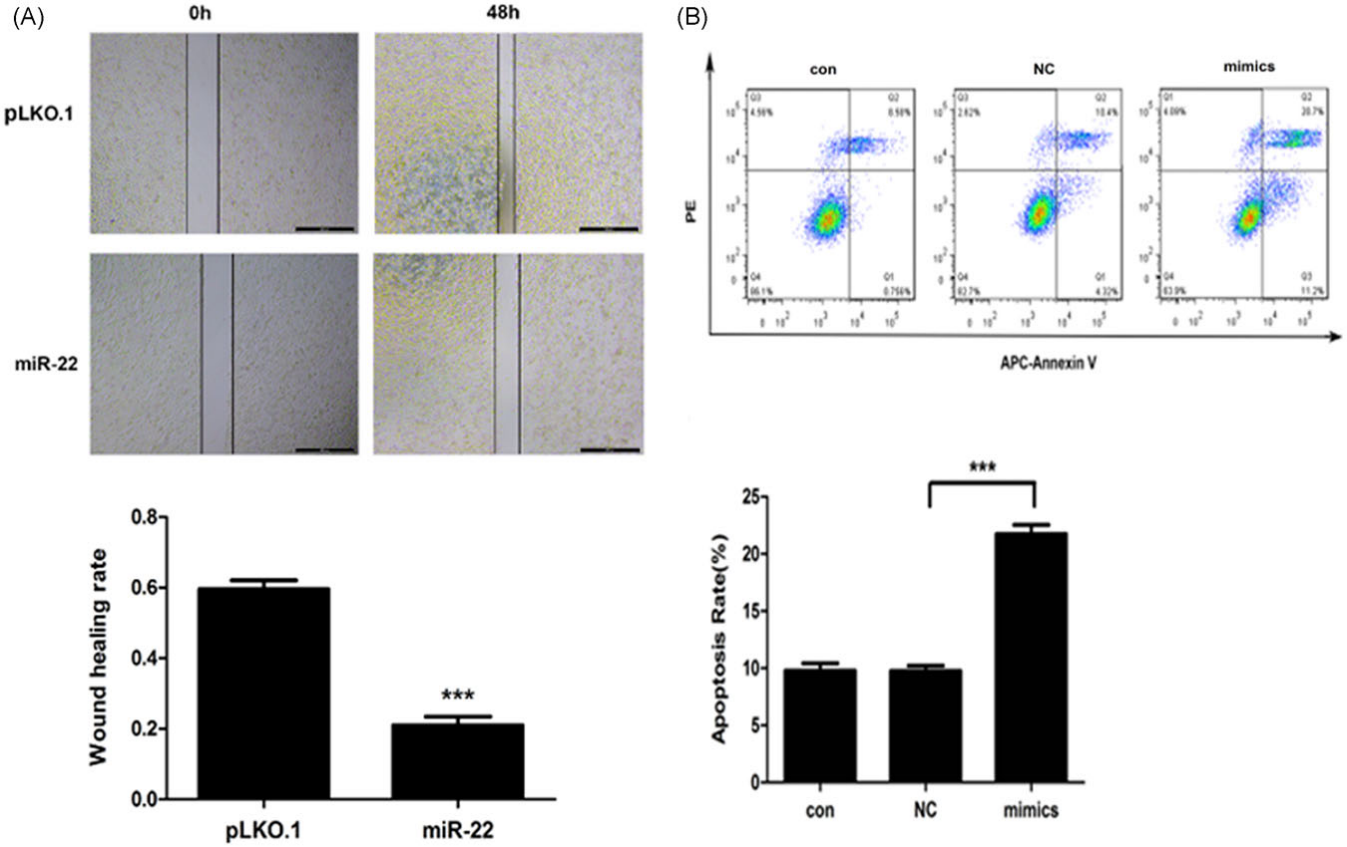
*miR‐22* can inhibit the cell migration and promote the cell apoptosis in SCLC (A, B) Cells were cultured in 96‐well plates for the indicated conditions, wound healing rate was determined by measurement, apoptosis was determined by flow cytometry analysis. *,**,***Significant differences between the tested and the control strains, *P* < .05, *P*  < .01, and *P*  < .001, respectively. SCLC, small‐cell lung cancer

To explore the effect of *miR‐22* on SCLC cell migration, we carried out the scratching trial. The results showed that the migration ability of *miR‐22* overexpression obviously decreased compared to control, revealing that *miR‐22* overexpression could significantly inhibit SCLC cell migration (Figure 6A).

*miR‐22*, as a tumor suppressor, can cause cell apoptosis to some extent.^20^ As a result, we next explored the effect of *miR‐22* on SCLC cell apoptosis by APC Annexin V/PI double staining. The results showed that the apoptosis rate of the *miR‐22* mimic transfection group was significantly higher than in the NC group (Figure 6B), illustrating that *miR‐22*, as a potential tumor suppressor, could induce SCLC cell apoptosis.

### 3.3 *miR‐22* is a negative regulator of WRNIP1 expression

**Figure 7:**
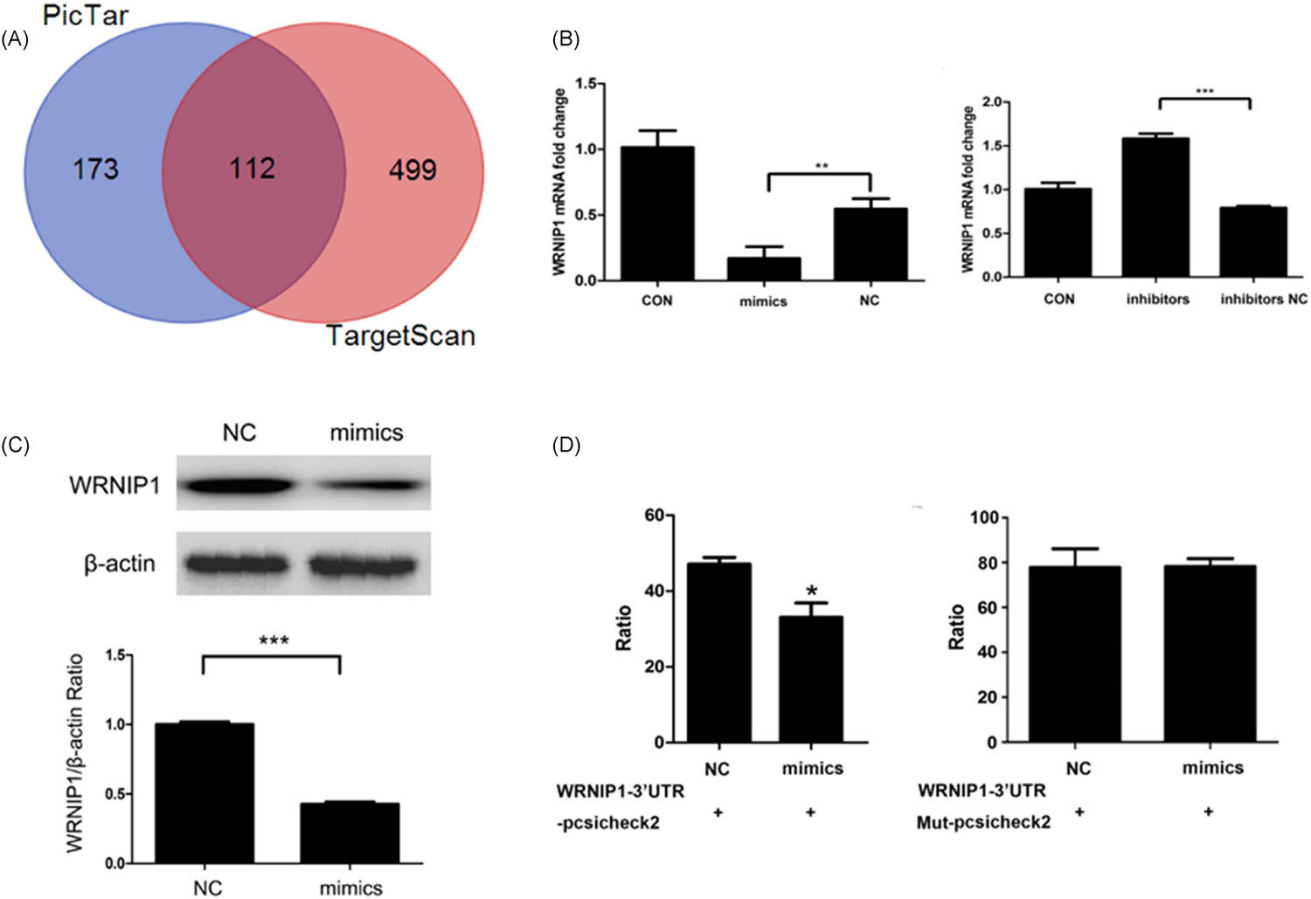
*miR‐22* can negatively regulate the *WRNIP1* expression in SCLC. A, Samples were cultured in the corresponding medium for the indicated conditions. Orange represents screening for the targeted genes by TargetScan, blue represents screening for the targeted genes by PICTAR, red represents screening for the overlapped genes by two databases. B, Cells were cultured in the corresponding medium for the indicated conditions. mRNA level was determined by quantitative PCR and measured as the ratio of *WRNIP1* relative to the internal reference target *β‐actin* gene. C, Cells were cultured in the corresponding medium for the indicated conditions. Protein level was determined by western blotting, and measured as the ratio of *WRNIP1* relative to the internal reference target *β‐actin* protein. D, Cells were cultured in the corresponding medium for the indicated conditions. Luciferase ratio was determined by microplate reader detection. *, **, ***Significant differences between the tested and the control strains, *P* < .05, *P*  <  .01, and *P*  <  .001, respectively. mRNA, messenger RNA; PCR, polymerase chain reaction; SCLC, small‐cell lung cancer

To further explore the mechanism of *miR‐22* affecting SCLC radiosensitivity, we predicted the target genes of *miR‐22* and preliminary screened the delineated genes by bioinformatics analysis. The results showed that the TargetScan database predicted 611 target genes and the PICTAR database predicted 285 ones. From Figure 7A, we can see 112 common target genes were predicted by both of the above databases. Through NCBI and a literature search, *WRNIP1*, as a candidate target gene, was found to be associated with DNA damage repair, which is helpful in elucidating the mechanism of *miR‐22* in SCLC cell radiosensitivity. Therefore, we selected *WRNIP1* for further research.

Therefore, we determined whether *miR‐22* negatively regulated *WRNIP1* during γ‐ray irradiation. Thus, we utilized the *miR‐22* mimics and inhibitors and compared these with the NC control in regard to transcription and translation levels. Collectively, these results indicated that *miR‐22* inhibits *WRNIP1* expression by qPCR and Western blotting (Figure 7B,C). It can be seen there may be a negative regulation relationship between *miR‐22* and *WRNIP1*, and *WRNIP1* may be a downstream target gene of *miR‐22*.

To confirm our conclusion, we next detected the luciferase activity in each group. The luciferase activity was significantly lower than in the NC group in *miR‐22* mimics and *WRNIP1*‐3′‐UTR‐psiCHECK2 double‐transfection. The luciferase activity in cells with the *miR‐22* mimics and *WRNIP1*‐3′‐UTR‐mut‐psiCHECK2 showed almost no significant difference from the NC group (Figure 7D), elucidating that *miR‐22* could be bound to the *WRNIP1‐*3′UTR. We eventually confirmed that *WRNIP1* is the direct downstream target gene of *miR‐22*. Furthermore, it was speculated that *miR‐22* was likely to enhance SCLC cell radiosensitivity by inhibiting *WRNIP1* expression.

**Figure 8:**
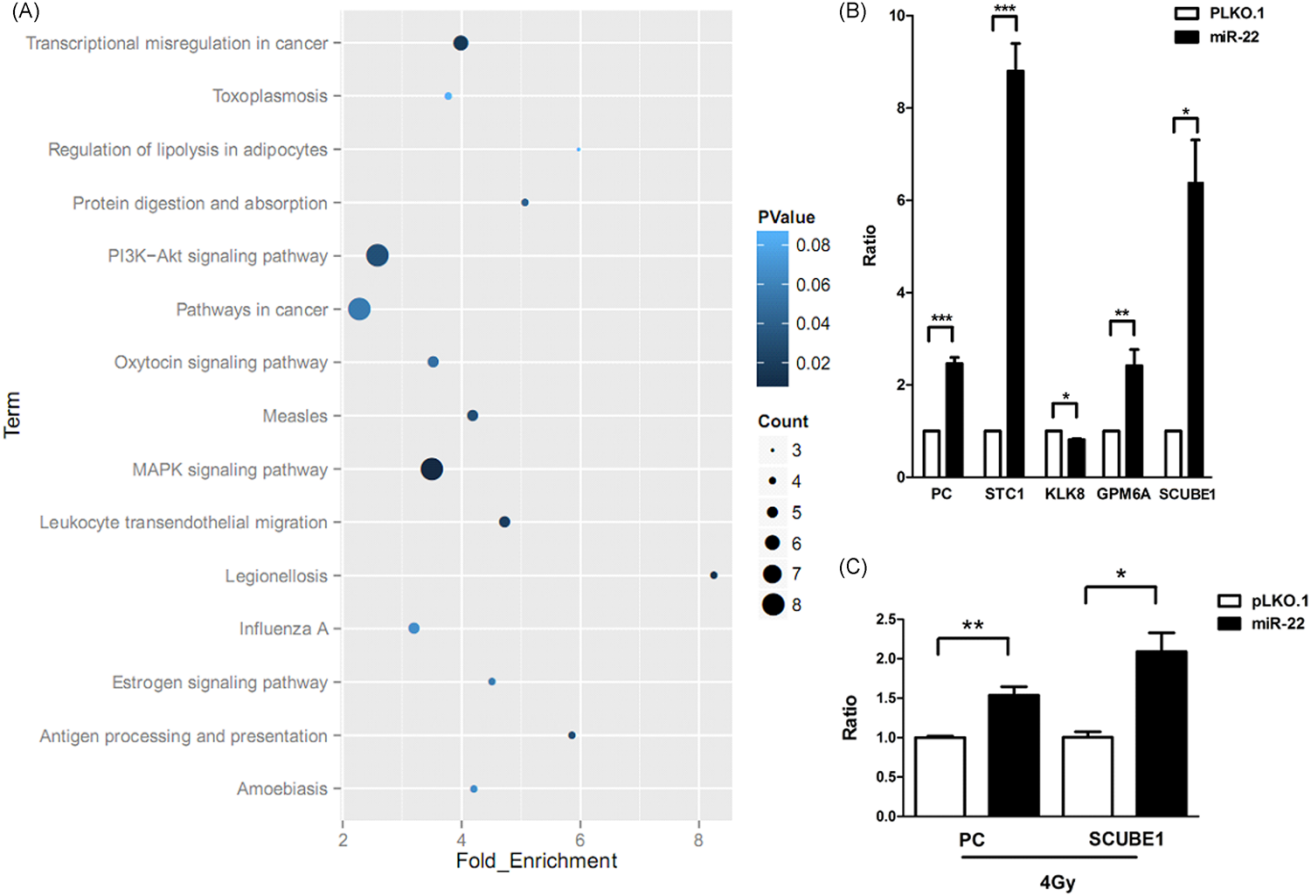
*miR‐22* changes the tumor‐related genes expression in SCLC after γ‐irradiation. A, Samples were cultured in the corresponding medium for the indicated conditions. *P* value represents the clustering DEGs analysis. Cells were cultured in the corresponding medium for the indicated conditions. mRNA level was determined by quantitative PCR and measured as the ratio of *PC* and *SCUBE1* relative to the internal reference target *β‐actin* gene. B, Cells were cultured in the corresponding medium for the indicated conditions. mRNA level was determined by quantitative PCR and measured as the ratio of *PC, STC1, KLK8, GPM6A*, and *SCUBE1* relative to the internal reference target β‐actin gene. C, Cells were cultured in the corresponding medium for the indicated conditions. mRNA level was determined by quantitative PCR and measured as the ratio of *PC* and *SCUBE1* relative to the internal reference target *β‐actin* gene. *, **, ***Significant differences between the tested and the control strains, *P* < .05, *P*  < .01, and *P*  < .001, respectively. DEG, differentially expressed genes; MAPK, mitogen‐activated protein kinases; mRNA, messenger RNA; PCR, polymerase chain reaction; P13K, phosphoinositide 3‐kinases; SCLC, small‐cell lung cancer

Moreover, we screened the high‐throughput DEGs *KLK8, PC, STC1, GPM6A*, and *SCUBE1*, which are related to cell proliferation, migration, and apoptosis, to detect mRNA levels (Figure 8A). qPCR was performed using the *miR‐22* overexpression transfected cell line and its blank control cDNA as template. The results showed that *KLK8* was significantly downregulated, and the other four genes were significantly upregulated upon overexpressing *miR‐22* (Figure 8B).

Under nonirradiation conditions, these five genes showed significant differences in transcription level, but their expression changed after γ‐irradiation. Only the changes of *PC* and *SCUBE1* showed consistency in the nonirradiation and γ‐irradiation conditions, although the increasing trend was weakened (Figure 8C). In summary, the expression of these five genes are related to γ‐ray irradiation.

In conclusion, miRNAs are key participants and regulators in cancer treatment. *miR‐22* can regulate tumor‐related gene expression, and it has a significant impact on LC diagnosis and treatment. Compared with the other common types of cancer, LC has a lower survival rate, which makes it the leading cause of cancer deaths worldwide. Tumor suppressors and carcinogenic factors miRNA are closely related to LC cell growth, development, and metastasis by changing the expression level. Otherwise, miRNAs are essential to the radioresistance and chemoresistance in LC. Furthermore, miRNAs also play an important role in cancer regulation. As a result, miRNAs may be used in LC clinical diagnosis, treatment, and prognosis in the future. With regard to miRNAs and cancer research, more samples and trials are needed to reveal the function of miRNAs in cancer research. Scientists have to further study the relationship between miRNAs and tumor radiosensitivity. As a result, miRNAs will become a promising tool for tumor prevention and treatment.

## 4 DISCUSSION

Radiotherapy is one of the main treatments for tumors.^21^ The efficacy of radiotherapy is affected by the sensitivity of tumor cells to radiotherapy.^22^ The radiosensitivity of different individual tumors is significantly different.^23^ Recently, with the in‐depth study of miRNAs, radiation conditions can induce changes in the expression of miRNAs, and some miRNAs participate in the regulation of tumor radiosensitivity by regulating the expression of target genes and vital signaling pathways.^24^ Shi et al^7^ reported that *miR‐200c* enhances the sensitization of LC cells A549 to radiation by targeting the VEGF‐VEGFR2 pathway. Hu et al^4^ performed qPCR detection for the expression of miRNAs in 102 patients with cervical cancer undergoing standardized treatments. They identified five miRNAs, *miR‐9, miR‐21, miR‐200a, miR‐218*, and *miR‐203*, that may be associated with cervical cancer radiosensitivity.

In the present study, we showed that the *miR‐22* expression in NCI‐H446 was significantly lower than that in BEAS‐2B. It has previously been reported that *miR‐22* plays an important role in multiple types of cancer development.^25^ Ling et al^15^ reported that *miR‐22* expression in human LC tissues was significantly decreased compared to normal control tissues. Compared with the normal ovarian tissue, Wyman et al^17^ found that the *miR‐22* expression was downregulated in ovarian cancer. Reports on gastric cancer also showed that *miR‐22* expression was rare in gastric cancer samples.^26^ However, there are few studies on *miR‐22* in SCLC cells.

In our study, we studied the biological function of *miR‐22* in an SCLC model for tumor progression and metastasis. Combined with the results of MTS and colony‐forming assays, we infer that *miR‐22* has a significant inhibitory effect on tumor growth and proliferation in SCLC cells. According to previous research, researchers found that *miR‐22* acted as a tumor suppressor, which had an inhibitory effect on the development of tumors.^27^, ^28^ Yang et al^19^ reported that *miR‐22* can significantly inhibit the cell proliferation, migration, and invasiveness of esophageal squamous cancer cells. Others found that *miR‐22* overexpression in ovarian granulosa cells could aggravate apoptosis and inhibit cancer cell growth.^29^

*Ki‐67* is an antigen expressed in proliferating cells and a common marker of cell proliferation; thus, it has been used as the most reliable indicator of tumor cell proliferation.^30^ We detected *Ki‐67* expression in *miR‐22* mimic‐ and inhibitor‐transfected cells and then explored the effect of *miR‐22* on apoptosis in SCLC by APC Annexin V/PI staining. These results indicated that *miR‐22*, as a tumor suppressor, could change cell proliferation and promote apoptosis in SCLC cells, and the results of our study were consistent with the previous outcome. In subsequent studies, we also need to detect the effects of *miR‐22* on the expression of apoptosis‐related proteins (*Bcl‐2, Bax*, and *Caspase‐3*)^15^ to further explain the specific molecular mechanism of *miR‐22* promoting cell apoptosis in SCLC.

Leuzzi et al^9^ revealed that *WRNIP1* can act on intracellular arrested replication forks and cooperate with *RAD51* to protect the integrity of replication forks. In the experiment, *WRNIP1*‐deficient cells exhibited significant DNA damage and chromosomal aberrations. Therefore, *WRNIP1* is considered as a protector of replication forks. Another publication suggests that *WRNIP1* can recruit DNA polymerase to DNA damage, which plays an important role in DNA damage repair and genome stability maintenance.^22^ Radiotherapy can cause severe, irreparable DNA damage and cell cycle arrest, leading to tumor death and cell apoptosis.^19^ It is speculated that *miR‐22* is likely to increase the radiosensitivity of SCLC cells by inhibiting *WRNIP1* expression.

The psiCHECK2 vector utilizes *Renilla* luciferase as a reporter gene, and the target gene is cloned into a multiple cloning site, which is downstream of the *Renilla* luciferase translation termination codon. Additionally, the psiCHECK2 vector contains a second reporter gene, firefly luciferase, which was designed for end‐point cleavage assays to normalize *Renilla* luciferase expression, resulting in robust and reproducible data.^11^, ^15^ Since the psiCHECK2 vector contains two kinds of luciferases, it can reduce the internal reference luciferase plasmid introduction during the experiment, thereby avoiding the multiplasmid cotransfection systems emergence and providing convenience for cell research.^13^ In addition, the *Renilla* luciferase assay is more sensitive, more convenient, and more rapid in quantification.^21^ The experimental results show that *miR‐22* can inhibit the reporter gene‐vector luciferase activity, indicating that *WRNIP1* is a direct target gene downstream of *miR‐22*.

In this study, to elucidate the mechanism of *miR‐22* affecting the radiosensitivity of SCLC cells, we analyzed the transcriptome using the *miR‐22* overexpression and control samples. Bioinformatics analysis highlighted five DEGs (*KLK8, PC, SCUBE1, STC1*, and *GPM6A*) in *miR‐22* overexpression and vector control cells, which were selected for quantitative detection.

*STC1* is a glycoprotein found in the endocrine glands of the fish kidney, and it is considered a tumor cell apoptosis‐inducing factor.^17^ *GPM6A* is a transmembrane protein widely distributed on the neuronal cells surface in the central nervous system; it is believed that *GPM6A* is one of the pathogenic genes of human lymphatic leukemia and is closely related to apoptosis.^13^ Therefore, we chose *STC1* and *GPM6A* for RT‐qPCR analysis. The results showed that *STC1* and *GPM6A* expression in the *miR‐22* overexpression cell line was significantly higher than that in the control group, which indicated that *STC1* and *GPM6A* could be the apoptosis‐inducing factors in SCLC cells. Therefore, *miR‐22* is likely to promote SCLC cell apoptosis by elevating the expression of apoptosis‐inducing factors *STC1* and *GPM6A* in tumors.

The kallikrein‐related peptidase enzyme (KLK) family, located on human chromosome 19q13.4, is a serine subfamily. *KLK8* is a member of this family, and abnormalities in *KLK8* transcription or translation products may lead to the development of uterine, ovarian, and other cancers.^25^ Therefore, it is speculated that *KLK8* might be a cancer‐promoting factor. In this study, we found that *KLK8* expression in *miR‐22* overexpression cells was significantly lower than another group, indicating that *KLK8* may be a cancer‐promoting factor and negatively regulated by *miR‐22*. Therefore, we further speculate that *miR‐22* inhibition of SCLC cell proliferation and migration may be related to the inhibition of *KLK8* expression.

*SCUBE1* is a glycoprotein secreted by platelet endothelium. Tokuda et al^27^ found that *SCUBE1* had an inhibitory effect on tumor development to some extent.^28^ Our results show that *SCUBE1* expression in *miR‐22* overexpression samples is higher than in the control, indicating that the inhibitory effect of *miR‐22* on tumor metastasis may be related to the upregulation of *SCUBE1* expression.

Pyruvate carboxylase (PC) is a kind of nonsteroidal enzyme, which is important to the tricarboxylic acid cycle.^6^ In addition, there is evidence that *PC* is associated with tumor invasiveness and metastasis.^14^ In our study, we found that *PC* was significantly increased in *miR‐22* overexpression cells, indicating that *miR‐22* could inhibit SCLC cell migration by regulating *PC* expression.

Because miRNAs can negatively regulate target genes, the results show that *STC1, GPM6A, PC*, and *SCUBE1* are also overexpressed in *miR‐22* overexpression cells, so it is presumed that these genes are not the direct target genes of *miR‐22*. In addition to the changes in the expression of the five DEGs, it is important to detect the expression of *STC1, GPM6A, PC, KLK8*, and *SCUBE1* in *miR‐22* overexpression cells under γ‐irradiation conditions. The results showed that only the changes in *PC* and *SCUBE1* were consistent, but the trend was weakening. Therefore, this study preliminarily explains the mechanism of *miR‐22* effects on the radiosensitivity of SCLC cells, and the follow‐up needs more in‐depth study.

## 5 CONCLUSION

At present, although independent studies have shown that miRNAs can be used to evaluate the radiosensitivity of tumors, which can guide the development of clinical radiotherapy, this approach is seldom applied clinically. Therefore, in the follow‐up study, we also need to study the expression of *miR‐22* in patients with SCLC undergoing radiotherapy and further explain the mechanism of *miR‐22* regulation of radiosensitivity in SCLC cells. Thus, improving the curative effect of radiotherapy, reducing radiation injury and minimizing the side effects in patients will be possible. Summarily, *miR‐22* may be widely used in the future as a means to evaluate tumor radiosensitivity and as a prognostic biomarker for patients receiving clinical treatment.

## CONFLICT OF INTERESTS

The authors declare that there is no conflict of interests.
